# TGF-β regulates nerve growth factor expression in a mouse intervertebral disc injury model

**DOI:** 10.1186/s12891-021-04509-w

**Published:** 2021-07-23

**Authors:** Yuji Yokozeki, Kentaro Uchida, Ayumu Kawakubo, Mitsufumi Nakawaki, Tadashi Okubo, Masayuki Miyagi, Gen Inoue, Makoto Itakura, Hiroyuki Sekiguchi, Masashi Takaso

**Affiliations:** 1 Department of Orthopaedic Surgery, Kitasato University School of Medicine, 1‐15‐1 Minami‐ku Kitasato, Sagamihara City, Kanagawa Japan; 2 Shonan University of Medical Sciences Research Institute, Nishikubo 500, Chigasaki City, Kanagawa 253-0083 Japan; 3 Department of Laboratory Animal Science, Kitasato University School of Medicine, Sagamihara, Kanagawa Japan; 4 Department of Biochemistry, Kitasato University School of Medicine, 1-15-1 Minami-ku, Kitasato, Sagamihara City, Kanagawa 252-0374 Japan

**Keywords:** Transforming growth factor-β, Nerve growth factor, Intervertebral disc

## Abstract

**Background:**

Intervertebral disc (IVD) degeneration is a major cause of low back pain (LBP). Following disc injury, nerve growth factor (NGF) concentrations rise in IVDs, and anti-NGF therapy has been shown to attenuate LBP in humans. Increased levels of tumor necrosis factor-α (TNF-α) and transforming growth factor-β (TGF-β) in degenerative IVDs and in in vitro studies suggest that these factors promote NGF production. However, whether these factors regulate NGF in vivo remains unclear. Thus, we studied NGF regulation in a mouse model of IVD injury.

**Methods:**

After inducing IVD injury, we examined mRNA levels of *Tnfa*, *Tgfb*, and *Ngf* in IVDs from control and IVD-injured mice across 7 days. To do this, we used magnetic cell separation to isolate CD11b ( +) (macrophage-rich) and CD11b (-) (IVD cell-rich) cell fractions from injured IVDs. To study the effect of TNF-α on *Ngf* expression, we examined *Ngf* expression in injured IVDs from C57BL/6 J and *Tnfa*-knockout (KO) mice (C57BL/6 J background). To study the effect of TGF-β on *Ngf* expression, C57/BL6J mice were given an intraperitoneal injection of either the TGF-β inhibitor SB431542 or DMSO solution (vehicle) one and two days before harvesting IVDs.

**Results:**

mRNA expression of *Tnfa*, *Tgfb*, and *Ngf* was significantly increased in injured IVDs. *Tnfa* was predominantly expressed in the CD11b ( +) fraction, and *Tgfb* in the CD11b (-) fraction. *Ngf* expression was comparable between CD11b ( +) and CD11b (-) fractions, and between wild-type and *Tnfa*-KO mice at post-injury day (PID) 1, 3, and 7. SB431542 suppressed TGF-β-mediated *Ngf* expression and NGF production in vitro. Further, administration of SB431542 significantly reduced *Ngf* expression in IVDs such that levels were below those observed in vehicle-treated animals at PID3 and PID7.

**Conclusion:**

A TGF-β inhibitor reduced *Ngf* expression in a mouse model of IVD injury, suggesting that TGF-β may regulate NGF expression in vivo.

**Supplementary Information:**

The online version contains supplementary material available at 10.1186/s12891-021-04509-w.

## Background

Persistent low back pain (LBP) is a common chronic ailment [1, 2]. A major cause of LBP is the degeneration of intervertebral discs (IVDs) [3, 4]. According to recent reports, the IVDs of animal disc injury models and humans with herniated IVDs show elevated levels of nerve growth factor (NGF) [5, 6]. Moreover, anti‐NGF therapy induces analgesia and shows efficacy in patients with chronic LBP [7–9]. The mechanisms governing NGF regulation, however, remain unclear.

Previous studies have suggested that tumor necrosis factor-α (TNF-α) and transforming growth factor-β (TGF-β) may be regulators of NGF. TNF-α is a major proinflammatory cytokine with powerful proinflammatory activity and the ability to promote secretion of a range of proinflammatory mediators. TNF-α expression is elevated in degenerated compared to non-degenerated IVDs [10, 11]. TNF-α has been shown to stimulate NGF in mouse and human IVD cells, including nucleus pulposus (NP) and anulus fibrosus (AF) cells, in vitro [6, 12]. Meanwhile, TGF-β may protect IVD tissue during restoration by promoting matrix synthesis and suppressing matrix catabolism, cell loss, and inflammatory response. However, over-activation of TGF-β is harmful to IVDs, with high levels of TGF-β observed in degenerated IVDs in mice and humans [6, 13]. We previously reported that TGF-β stimulates NGF production in mouse IVD cells in vitro [6]. Accumulating evidence from in vitro studies thus suggests that TNF-α and TGF-β contribute to the regulation of NGF during IVD degeneration. However, whether TNF-α and TGF-β also regulate NGF expression in vivo remains unclear.

Here, we investigated the role of TNF-α and TGF-β in relation to NGF expression in a mouse IVD puncture model in vivo.

## Methods

### Animals

Male C57BL/6 J mice and *Tnfa*-knockout (KO) mice (C57B/6 J background) aged nine weeks were kept in an animal housing system maintained at 23 °C ± 2 °C and 55% ± 10% humidity under a 12‐hour light/dark cycle for the duration of the study. The experimental protocol was approved by the Kitasato University School of Medicine Animal Care Committee (reference number: 2020–089). The study was conducted according to the ARRIVE guidelines for the reporting of animal experiments. All methods complied with the guidelines for the proper conduct of animal experiments of the Science Council of Japan.

### Expression of *Tnfa*, *Tgfb*, and *Ngf* in an IVD injury mouse model

*Tnfa*, *Tgfb*, and *Ngf* expression was examined in 40 C57BL/6 J mice following IVD injury. Ten mice were randomly chosen to form the control group, while the remaining 30 mice formed the IVD injury group. After receiving anesthesia comprising isoflurane followed by an intramuscular injection of a 1:3:1 mixture of midazolam (Product no. KB6762, Sando, Yamagata, Japan), Domitor (medetomidine hydrochloride; Product no. 005130, Orion Corporation, Espoo, Finland), and Vetorphale (butorphanol tartrate; Product no. VETLI5, Meiji Seika Pharma Co., Ltd., Tokyo, Japan) into the upper limbs (0.05 ml/100 g body weight), mice in the IVD injury group were subjected to a puncture injury, where a 27-gauge needle was used to puncture IVDs (coccygeal discs 5–6 and 6–7) 10 times. Control mice were subjected to all parts of the surgery except for the puncture injury. *Tnfa*, *Tgfb*, and *Ngf* expression was subsequently examined on post-injury days 0 (PID0), 1 (PID1), 3 (PID3), and 7 (PID7) using quantitative polymerase chain reaction (qPCR; *n* = 10 per time point). NP and AF tissues were not separated for analysis.

### qPCR

IVDs were harvested and immediately homogenized in TRIzol (Product no. 15596026, Invitrogen, Carlsbad, CA) on ice; the digested sample was used as the template for first‐strand complementary DNA (cDNA) synthesis using SuperScript™ III RT (Product no. 18080085, Invitrogen). PCR reactions (25 μl) comprised 2 μl cDNA, a specific primer set (0.2 μM final concentration), and 12.5 μl SYBR Premix Ex Taq (Product no. RR820, Takara, Kyoto, Japan). Table 1 lists the primers used in this study. PCR product size was confirmed by gel electrophoresis using cDNA extracted from intact IVDs (Supplementary Figure 1). qPCR using a RT‐PCR Detection System (CFX‐96; Bio‐Rad, Hercules, CA) was performed at 95 °C for 1 min, followed by 40 cycles of 95 °C for 5 s, and 60 °C for 30 s. mRNA expression of the genes of interest was normalized to *Gapdh* expression. Relative expression was calculated based on the mean value from control samples (non‐injured discs from the control group or vehicle‐treated disc cells in vitro).

**Table 1:** Primer sequences

Primer	Sequence (5'–3')	Product size (bp)
*Tnfa-*F	TGGCAATTCAGGAGAGGCAG	109
*Tnfa-*R	AGTGGTTGGAGAAACAGGCA	
*Tgfb-*F	CTCCCGTGGCTTCTAGTGC	133
*Tgfb-*R	GCCTTAGTTTGGACAGGATCTG	
*Ngf*-F	ATGGTGGAGTTTTGGCCTGT	192
*Ngf*-R	GTACGCCGATCAAAAACGCA	
*F4/80*-F	TGGGATGTACAGATGGGGGA	189
*F4/80*-R	CCTGGGCCTTGAAAGTTGGT	
*Cd11b-*F	CTGGCTTTAGACCCTGTCCG	138
*Cd11b-*R	GTCCACGCAGTCCGGTAAAA	
*Col2a1-*F	GGAGAGACCATGAACGGTGG	78
*Col2a1-*R	CATCTGGACGTTAGCGGTGT	
*Gapdh*-F	AACTTTGGCATTGTGGAAGG	223
*Gapdh*-R	ACACATTGGGGGTAGGAACA	

### *Tnfa, Tgfb* and *Ngf* expression following IVD injury

We previously observed increased *Tnfa*, *Tgfb* and *Ngf* expression levels at PID7 in wild-type mice [6]. We also confirmed that > 90% of CD11b ( +) cells were F4/80^high^ cells (macrophages) at PID7 (Supplementary Figure 2). Therefore, we examined *Tnfa*, *Tgfb* and *Ngf* expression in CD11b ( +) cells (macrophages) and IVD cells isolated from IVDs on PID7. IVDs (*n* = 5) were digested in 0.25 mg/ml collagenase overnight at 37 °C. Thereafter, cells were passed through a cell strainer and then incubated with biotin-conjugated anti-CD11b antibody (clone M1/70, Product no.101204, BioLegend, CA, USA) for 30 min at 4 °C. The cells were washed twice in PBS before incubating with streptavidin-conjugated magnetic beads (Product no. 557812, BD Biosciences, San Diego, CA, USA) for 30 min at 4 °C. The cells were transferred to a magnetic board (Product no. 552311, BD Biosciences) and incubated for 6 min at room temperature. Unbound CD11b (-) cells (disc cells) were collected before removing the remaining cells from the magnetic board and collecting bound CD11b ( +) cells (macrophages). CD11b (-) and ( +) cells were centrifuged at 300 g for 5 min and dissolved in TRIzol. After cDNA synthesis, as described above, a 10 μl reaction mixture containing SuperMix, a reagent from Perfecta Preamp SuperMix (Product no. 95146–040, Quanta Biosciences, MA, USA), 5 μl primer pool (500 nM forward and 500 nM reverse), and 35 μl cDNA was prepared for preamplification PCR for 3 min at 95 °C, followed by 14 cycles of 15 s at 95 °C and 3 min at 60 °C. Pre-amplification PCR products were immediately diluted (1:16) and used for qPCR. Successful isolation of CD11b cells was determined based on *F4/80* and *Cd11b* mRNA expression in each fraction.

### Generation of *Tnfa* KO mice

We designed two guide RNAs (gRNA 5, 6) in exon 1 and exon 4, comprising 20-nucleotide sequences targeting exons 1–4 of the *Tnfa* gene (5′- GGAGGGAGATGTGGCGCCTT-3′ and 5′- GAGTCCGGGCAGGTCTACTT-3′). Constant regions of CRISPR RNA (crRNA) and trans-activating crRNA (tracrRNA) were synthesized by Thermo Fisher Scientific (Carlsbad, CA, USA). Electroporation into fertilized C57BL/6 J zygotes was performed based on a previously reported procedure [14]. After incubation of the zygotes, surviving two-cell-stage embryos were transferred to the oviducts of pseudo-pregnant female mice.

### Genotyping

**Fig. 1:**
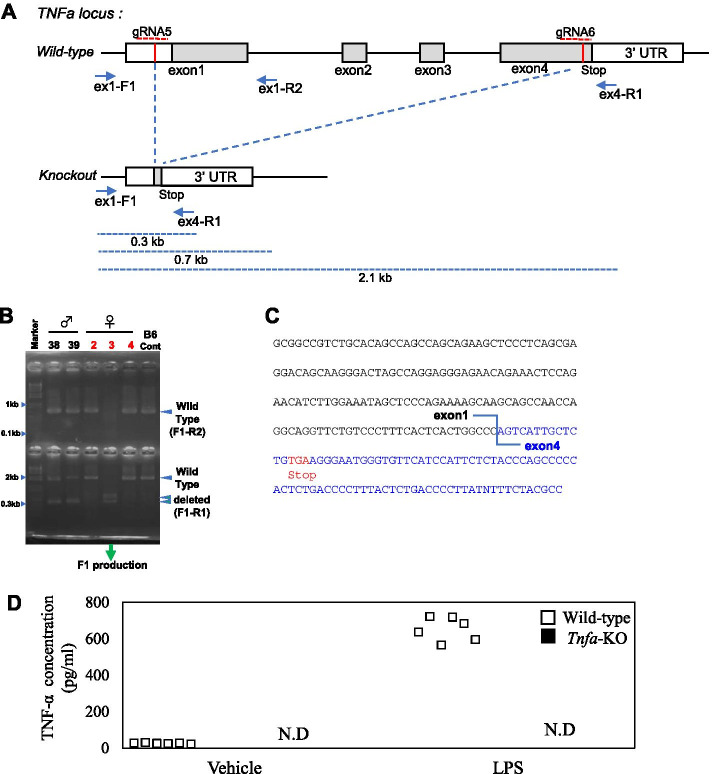
Generation of *Tnfa* knockout mice. **A** Schematic showing the structure and map of the mouse *Tnfa* locus. The location of the two gRNAs and primer sequences for polymerase chain reaction (PCR) are shown. **B** PCR screen for *Tnfa*-KO founder mice. **C** The gene sequence at the junction of the deleted alleles. **D** Confirmation of loss of TNF-α protein by ELISA (*n* = 6)

To assess CRISPR/Cas9-mediated deletion of the *Tnfa* gene, we performed PCR on tissue-extracted genomic DNA using specific primer sets for *Tnfa* KO mice (ex1-F1: 5′-TTCCTTGATGCCTGGGTGTC-3′, ex1-R2: 5′-TCCGAGGTCCTGACTCTGTCC-3′, and ex4-R1 5′-GTTAGAAGGATACAGACTGGG-3′). PCR was performed using Ex-Taq DNA polymerase (Product no. RR01, Takara Bio, Shiga, Japan) as follows: 35 cycles at 94 °C for 20 s, 61 °C for 10 s, and 72 °C for 20 s. The size of the deleted allele is 0.3 Kbp and that of the wild-type fragments are 0.7 Kbp and 2.1 Kbp (Fig. 1A, B).

### ELISA

We confirmed the absence of TNF-α protein in *Tnfa*-KO mice using ELISA. Bone marrow macrophages (BMMs) were harvested from wild-type and *Tnfa*-KO mice and exposed to 1 μg/ml lipopolysaccharide (LPS; Product no. ALX-581–017-L002, Enzo Life Sciences, Farmingdale, NY) for 24 h (*n* = 6). Concentrations of TNF-α in cell supernatants were determined using a commercial TNF-α ELISA kit (Product no. 430907, BioLegend).

### Effect of *Tnfa *deficiency on *Ngf* expression in IVD injury

Forty C57BL/6 J and *Tnfa*-KO mice were used to study the role of TNF-α in NGF expression. Ten mice were randomly selected to form the control group, while the remaining 30 formed the IVD injury group. IVD injury was induced as described above. *Ngf* expression were determined using qPCR (*n* = 10 per time point) on PID0, PID1, PID3, and PID7.

### Effect of TGF‐β inhibitor, SB431542, on NGF expression and production in IVD cell culture

To determine whether the TGF‐β inhibitor, SB431542, inhibits *Ngf* expression and NGF production in IVD cells, we examined the effect of SB431542 on TGF‐β-mediated *Ngf* expression and NGF production by IVD cells isolated from five mice. IVD cells were isolated using collagenase digestion, as described above. Disc cells were subsequently incubated in α‐minimal essential media (α‐MEM) with 10% fetal bovine serum in six‐well plates. One week later, IVD cells were stimulated with α‐MEM (vehicle), 10 ng/ml recombinant TGF‐β (Product no. 7666-MB, R&D Systems, Minneapolis, MN, USA), or 10 ng/ml recombinant TGF‐β + 1 µM SB431542 (Product no. S4317, Sigma Aldrich, St Louis, MO, USA) for 6 and 24 h. Thereafter, total mRNA was extracted and analyzed using qPCR. NGF concentration in the cell supernatant was determined using a commercial NGF ELISA kit (Product No. DY556, R&D Systems).

### Effect of a TGF-β inhibitor in IVD injury model mice

**Fig. 2:**
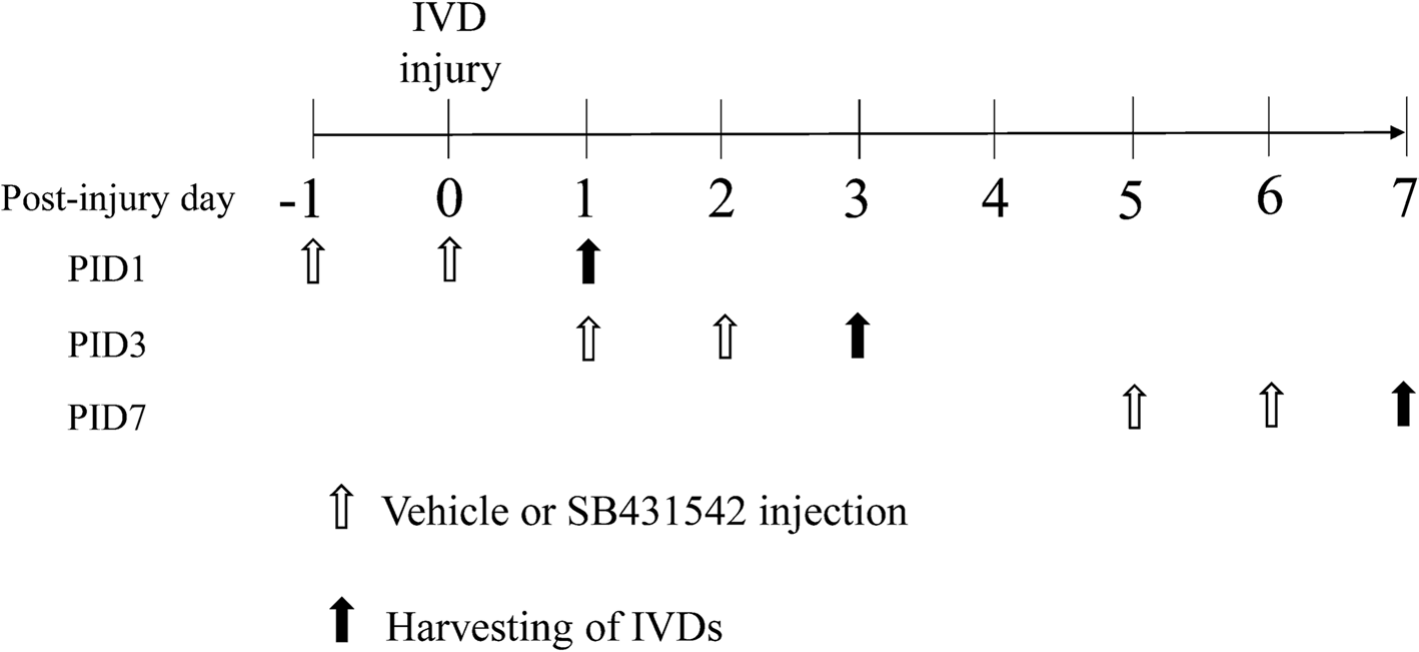
Administration scheme of transforming growth factor-β (TGF-β) inhibitor. SB431542 or DMSO (vehicle) was intraperitoneally injected into mice with intervertebral disc injury 1 and 2 days before harvesting intervertebral discs at post-injury day 1, 3, and 7

The 60 mice that received the IVD injury described above were randomly assigned to two equal groups: vehicle and treatment groups. The 30 mice in the treatment group received an intraperitoneal (IP) injection of 10 mg/kg SB431542 in 5% DMSO solution (SB43152) 1 and 2 days before harvesting IVDs, and the remaining 30 in the vehicle group received an IP injection of 5% DMSO solution (vehicle) at same time points (Fig. 2). Dosage was chosen according to the optimal inhibitory effect of an IP injection of a 10 mg/kg dose, as reported previously [13, 15]. IVDs were harvested at PID1, PID3, and PID7 and subjected to qPCR analysis (*n* = 10 for each time point).

### Statistical analysis

Statistical analyses were performed using SPSS software version 11.0 (SPSS Inc., Chicago, IL). Gene and protein expression was analyzed using one‐way analysis of variance followed by Bonferroni’s post hoc multiple comparisons test. After performing a Kolmogorov–Smirnov test, a Wilcoxon signed rank test or paired t-test was used to compare differences between CD11b ( +) and CD11b (-) fractions. Moreover, following a Kolmogorov–Smirnov test, t-test or a Mann–Whitney U test was used to compare differences between wild-type and *Tnfa*-KO mice or vehicle- and TGF-β inhibitor-treated groups at each time point. *P* < 0.05 was considered significant.

## Results

### *Tnfa, Tgfb,* and *Ngf* expression after IVD injury

**Fig. 3:**
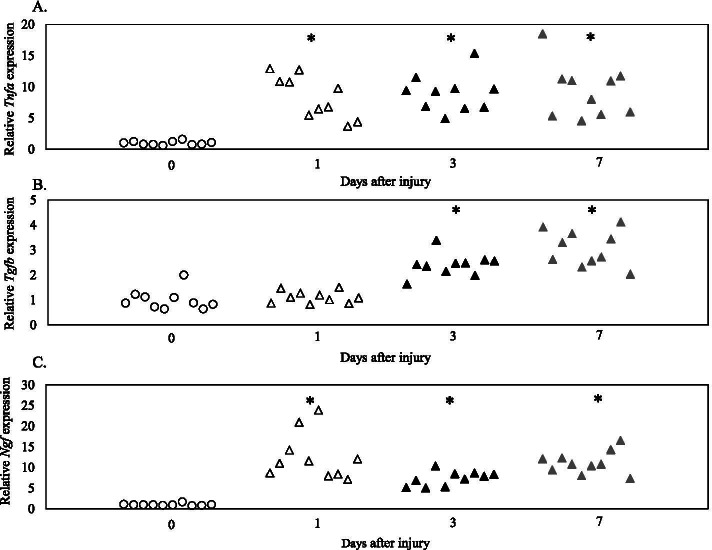
*Tnfa*, *Tgfb, Ngf* expression following IVD injury. Expression of *Tnfa* (**A**), *Tgfb* (**B**), and *Ngf* (**C**) at post-injury day 0, 1, 3, and 7 (*n* = 10 for each time point). *P < 0.05 compared to day 0 (control)

There was a significant increase in *Tnfa* expression at PID1, 3 and 7 (PID1, *P* < 0.001; PID3, *P* < 0.001; PID7, *P* < 0.001; Fig. 3A) and *Tgfb* expression at PID3 and 7 (PID1, *P* = 1.000; PID3, *P* < 0.001; PID7, *P* < 0.001; Fig. 3B) in wild-type mice. Similarly, *Ngf* expression was significantly increased at PID1, 3 and 7 (PID1, *P* < 0.001; PID3, *P* = 0.001; PID7, *P* < 0.001; Fig. 3C) in wild-type mice.

### *Tnfa*,* Tgfb* and *Ngf* expression in IVD cells

**Fig. 4:**
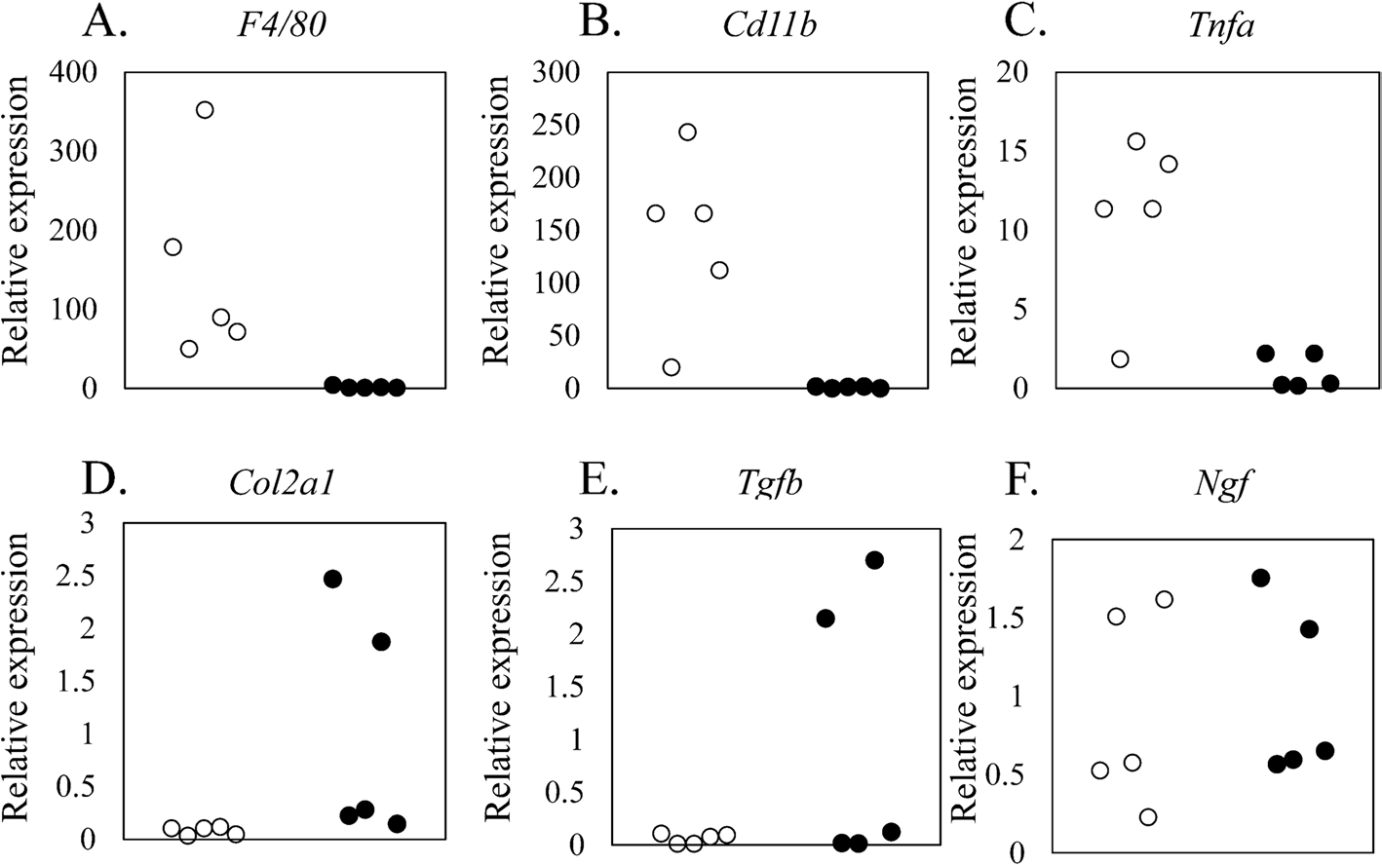
Gene expression in CD11b-positive and -negative cells following IVD injury. Expression of *F4/80* (**A**), *Cd11b* (**B**), *Tnfa* (**C**) *Col21a* (**D**), *Tgfb* (**E**), and *Ngf* (**F**) in CD11b ( +) and CD11b (-) cell fractions 7 days after IVD injury (*n* = 5). *P < 0.05

Between CD11b ( +) and (-) cells, *Cd11b*, *F4/80*, and *Tnfa* were predominantly expressed in CD11b ( +) cells (*F4/80*, *P* = 0.018; *Cd11b*, *P* = 0.018; *Tnfa*, *P* = 0.026; Fig. 4A–C). In contrast, *Col2a1* and *Tgfb* were predominantly expressed in CD11b (-) cells (*Col2a1*, *P* = 0.026; *Tgfb*, *P* = 0.026; Fig. 4D–E). Meanwhile, *Ngf* expression was comparable between CD11b ( +) and CD11b (-) cells (*P* = 0.459; Fig. 4F).

### Effect of *Tnfa* deficiency on *Ngf* expression following IVD injury

**Fig. 5:**
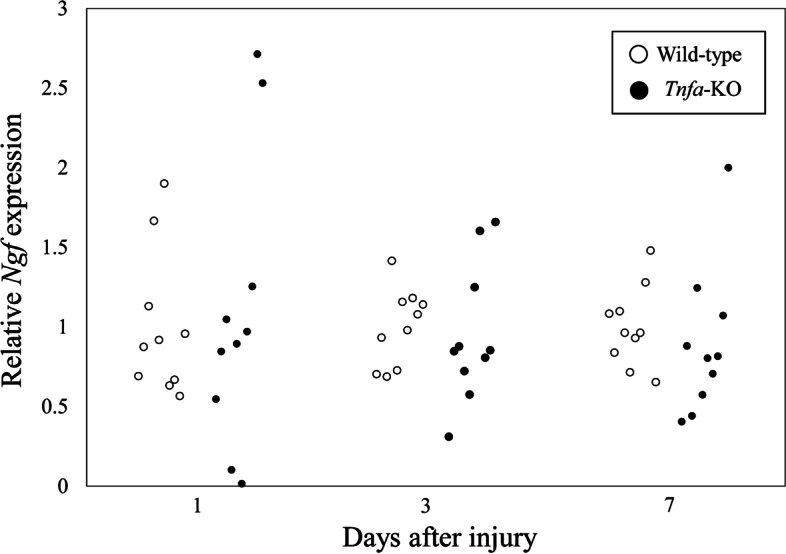
Effect of *Tnfa* deficiency on *Ngf* expression in vivo. Expression of *Ngf* 1, 3, and 7 days after IVD injury in wild-type (C57BL/6 J) and *Tnfa*-KO mice (*n* = 10 for each time point). Values represent mean ± standard error. *P < 0.05 compared to wild-type at the same time point. Relative *Ngf* expression was calculated based on *Ngf* expression in wild-type mice at each time point

To knock out the *Tnfa* gene, we used CRISPR/Cas9 to design two gRNAs to delete exons 1–4 (Fig. 1A). This method led to the successful generation of three independent deletion mutant mouse founders (Fig. 1B). From these, we established an F1 population by crossing with C57BL/6 J mice and checking the genotype of the offspring. We confirmed that all offspring had deletions in exons 1–4 (Fig. 1C). LPS stimulation of BMMs derived from wild-type mice significantly increased supernatant TNF-α levels (*P* < 0.001; Fig. 1D). However, TNF-α protein levels in vehicle- and LPS-stimulated BMMs derived from *Tnfa*-KO mice remained below the detection limit (< 7.8 pg/ml). To evaluate the effect of *Tnfa* on *Ngf* expression, we compared *Ngf* expression after IVD injury in wild-type and *Tnfa*-KO mice. *Ngf* expression was comparable between wild-type and *Tnfa*-KO mice at all time points examined (PID1, *P* = 0.842; PID3, *P* = 0.578; PID7, *P* = 0.053; Fig. 5).

### Effect of a TGF-β inhibitor on IVD cell culture

**Fig. 6:**
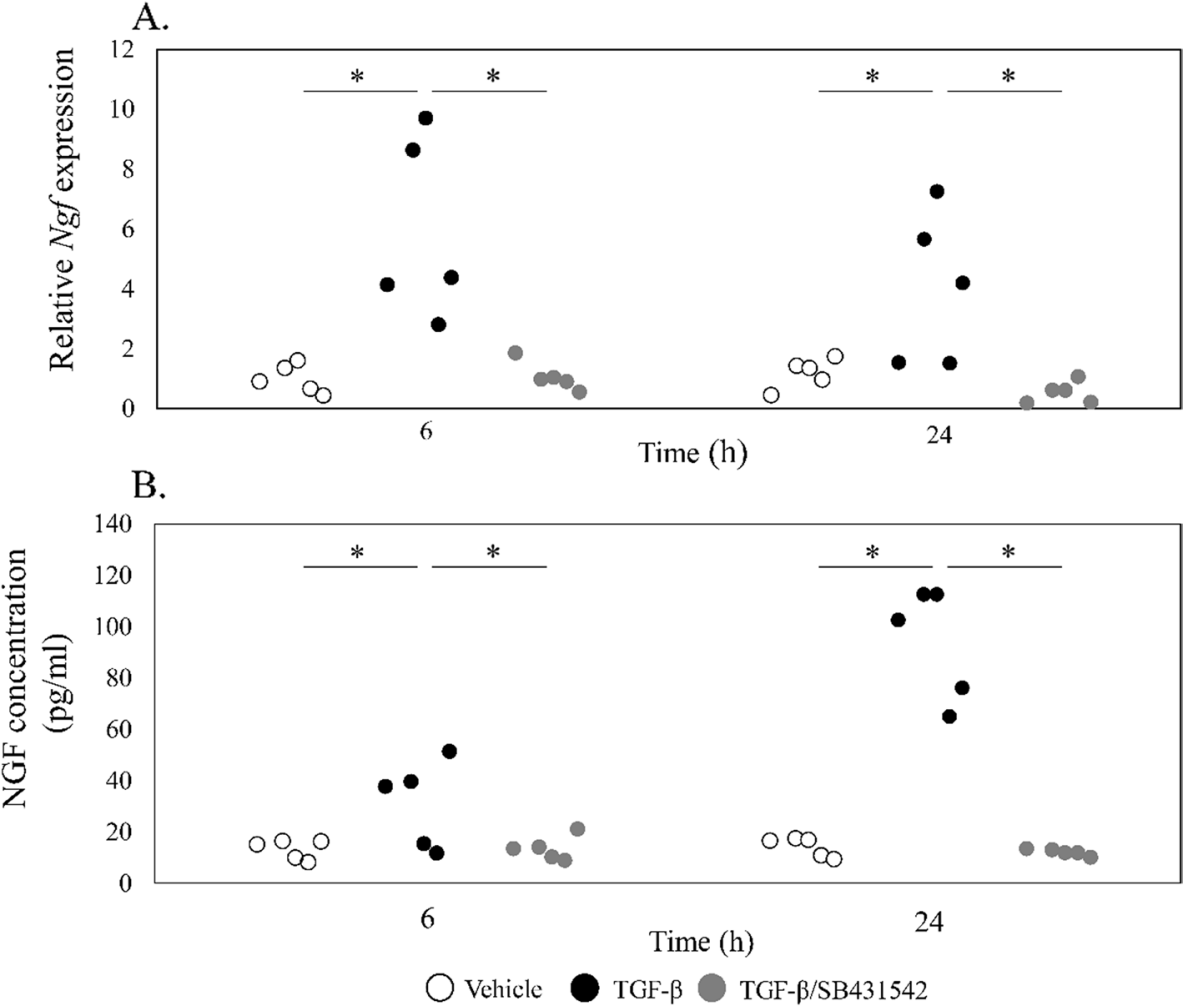
Effect of TGF-β and a TGF-β inhibitor on *Ngf* expression and NGF production in vitro. **A** RT-PCR for *Ngf*. Disc cells were incubated with α-MEM control, TGF-β, or TGF-β/SB431542 for 6 and 24 h. Relative expression was calculated based on expression in control samples. **B** ELISA for NGF. Disc cells were incubated with α-MEM control, TGF-β, or TGF-β/SB431542 for 6 and 24 h. *P < 0.05 compared to control

Stimulation of IVD cells with TGF-β for 6 and 24 h significantly increased *Ngf* mRNA expression (6 h, *P* = 0.013; 24 h, *P* = 0.038; Fig. 6A), and addition of the TGF-β inhibitor SB431542 completely reversed this effect (6 h, *P* = 0.025; 24 h, *P* = 0.040; Fig. 6A). Likewise, stimulation with TGF-β for 6 and 24 h increased supernatant NGF protein levels compared to vehicle (6 h, *P* = 0.049; 24 h, *P* = 0.001; Fig. 6B), and addition of SB431542 completely reversed this effect (6 h, *P* = 0.036; 24 h, *P* = 0.002; Fig. 6B).

### Effect of a TGF-β inhibitor in an IVD injury model

**Fig. 7:**
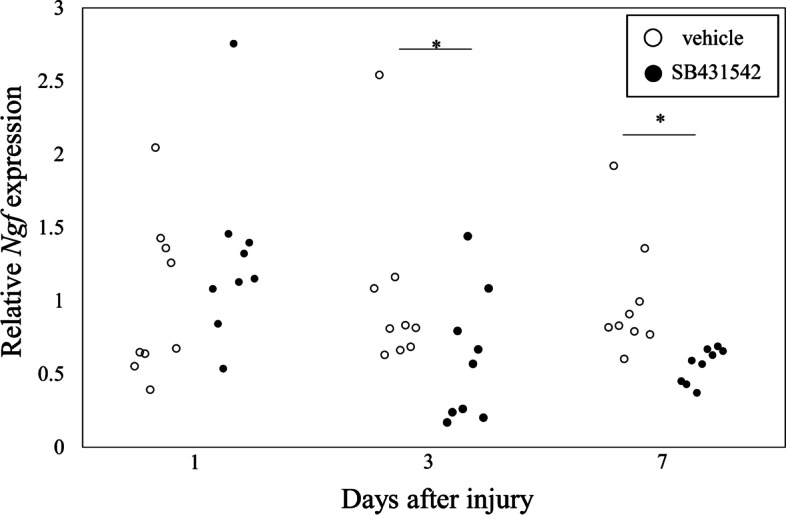
Effect of a TGF-β inhibitor on *Ngf* expression in vivo. SB431542 or DMSO (vehicle) was intraperitoneally injected into mice with intervertebral disc injury 1 and 2 days before harvesting intervertebral discs at post-injury day 1, 3, and 7 (*n* = 10 for each time point). *P < 0.05 compared to vehicle. Relative *Ngf* expression was calculated based on *Ngf* expression in the vehicle-treated group at each time point

Injection of the TGF-β inhibitor SB431542 into mice at PID1 produced no difference in *Ngf* expression compared to the vehicle-treated group (*P* = 0.354; Fig. 7). In contrast, injection of SB431542 into mice at PID3 and PID7 led to a significant reduction in *Ngf* expression compared to the vehicle-treated group (*P* = 0.038, *P* = 0.0001, respectively).

## Discussion

Several studies have generated IVD injury models by performing tail puncture under varying conditions [6, 16–22]. A model in which puncture injury was induced using a 31-gauge needle showed a gradual increase in degenerative score and decrease in glycosaminoglycan levels in IVDs at 6 and 12 weeks post-puncture [22]. A model in which puncture injury was induced using a 26-gauge needle displayed lower disc height 8 weeks post-puncture than that in the control group, while a model generated using a 29-gauge needle showed comparable disc height [17]. We generated an IVD injury model by performing 10 punctures with a 27-gauge needle and found that this model was useful for evaluating increased inflammatory cytokine and growth factor levels, and macrophage recruitment across a short time period of 1 to 28 days. [6, 16, 18, 19] Therefore, we used this model to evaluate the effects of TNF-α and TGF-β on NGF expression.

A number of reports have demonstrated that NGF levels rise locally at sites of inflammation [23–26]. TNF-α is a major factor in IVD inflammation [11], and has been shown to promote NGF expression and production in mouse IVD cells [6] and human AF and NP cells in vitro [12]. Therefore, TNF-α is thought to regulate NGF in IVD injury. However, we found that although *Tnfa* and *Ngf* expression was immediately elevated in injured IVDs in wild-type mice at PID1, *Tnfa* deficiency did not alter *Ngf* expression in injured IVDs. In our IVD puncture model, *Tnfa* was predominantly expressed in macrophages in injured IVDs. We previously reported that depletion of macrophages using clodronate liposomes in a mouse IVD injury model, generated using 10 punctures with a 27-gauge needle, reduced *Tnfa*, but not *Ngf* expression at PID1 [18]. In addition, while we also found that exposure of human synovial fibroblasts and macrophages to recombinant TNF-α led to elevated *NGF* expression and NGF production in vitro, *NGF* levels did not correlate with *TNFA* expression levels in human synovial tissue [27]. Thus, while TNF-α levels may rise in IVDs as a result of an increase in the macrophage population, elevated TNF-α levels may not play a major role in regulating NGF expression after IVD injury.

Previous studies have reported elevated levels of TGF-β in degenerated IVDs and in chondrocyte-like cells compared to fibroblastic cells in IVDs [6, 28]. However, TGF-β can also be produced by macrophages under pathogenic conditions [29, 30]. Macrophages in the kidneys of a kidney injury mouse model express higher levels of TGF‐β in the recovery phase [29]. Similarly, macrophages express TGF‐β in an adriamycin-induced nephrosis mouse model [30]. We compared TGF-β expression in disc cells and macrophages isolated from IVDs and found that TGF-β was predominantly expressed in IVD cells. Further, TGF-β stimulation increased NGF production by IVD cells in vitro and, administration of a TGF-β inhibitor reduced *Ngf* expression in vivo in an IVD injury mouse model. Our results indicate that TGF-β regulates NGF during IVD degeneration and that changes in *Ngf* expression may be due to increased autocrine/paracrine activity of IVD cell-derived TGF-β.

There were several limitations in this study. First, neither the TGF-β inhibitor nor *Tnfa* deficiency suppressed elevated *Ngf* expression on PID1, suggesting there may be other regulators of NGF in the acute phase. Further investigation is needed to identify these other NGF regulators. Second, previous studies have reported that *Tnfa-*KO mice do not exhibit an obvious phenotype due to functional redundancy [31] or rewiring of genetic networks [32]. Further investigation is needed to determine the precise mechanisms underlying TNF-α-mediated regulation of *Ngf* expression in vivo. Third, as we performed 10 needle punctures to induce major injury, other cells besides macrophages (such as endothelial cells) may have migrated into IVDs from the surrounding tissue, and contributed to the observed effects. Finally, we could not separate NP and AF cells due to the major injury induced as a result the 10 needle punctures.

## Conclusion

A TGF-β inhibitor reduced *Ngf* expression in an IVD injury mouse model, suggesting that TGF-β may regulate NGF expression in vivo.

## Supplementary Information

**Additional file 1.**

**Additional file 2.**

## Data Availability

Datasets supporting the conclusions of this article are included within the article. The raw data can be requested from the corresponding author. Data are submitted to DDBJ Sequence Read Archive under accession number LC636331.

## Abbreviations

IVD: Intervertebral disc
TNF-α: Tumor necrosis factor-α
TGF-β: Transforming growth factor-beta
LBP: Low back pain
NGF: Nerve growth factor
PCR: Polymerase chain reaction
GAPDH: Glyceraldehyde-3-phosphate dehydrogenase
