# Supplementary material for: TGF-β regulates nerve growth factor expression in a mouse intervertebral disc injury model
Source: BMC Musculoskelet Disord. 2021 Jul 23;22:634. doi: 10.1186/s12891-021-04509-w (PMC8299674; doi:10.1186/s12891-021-04509-w)
Supplement: Supplementary file 1 — Additional file 1. [file 12891_2021_4509_MOESM1_ESM.pdf]

Supplementary material 1

To confirm successful amplification of quantitative polymerase chain reaction (qPCR) products, amplified products from cDNA samples extracted from intact discs were examined by electrophoresis on a 3% agarose gel stained with ethidium bromide and visualized using FAS-IV (Nippon Genetics Co., Ltd., Tokyo, Japan). A 100-bp DNA ladder (Product no. 3407A, Takara Bio, Shiga, Japan) was used as the size standard. Single bands appear at the predicted product size (Supplemental Figure 1).

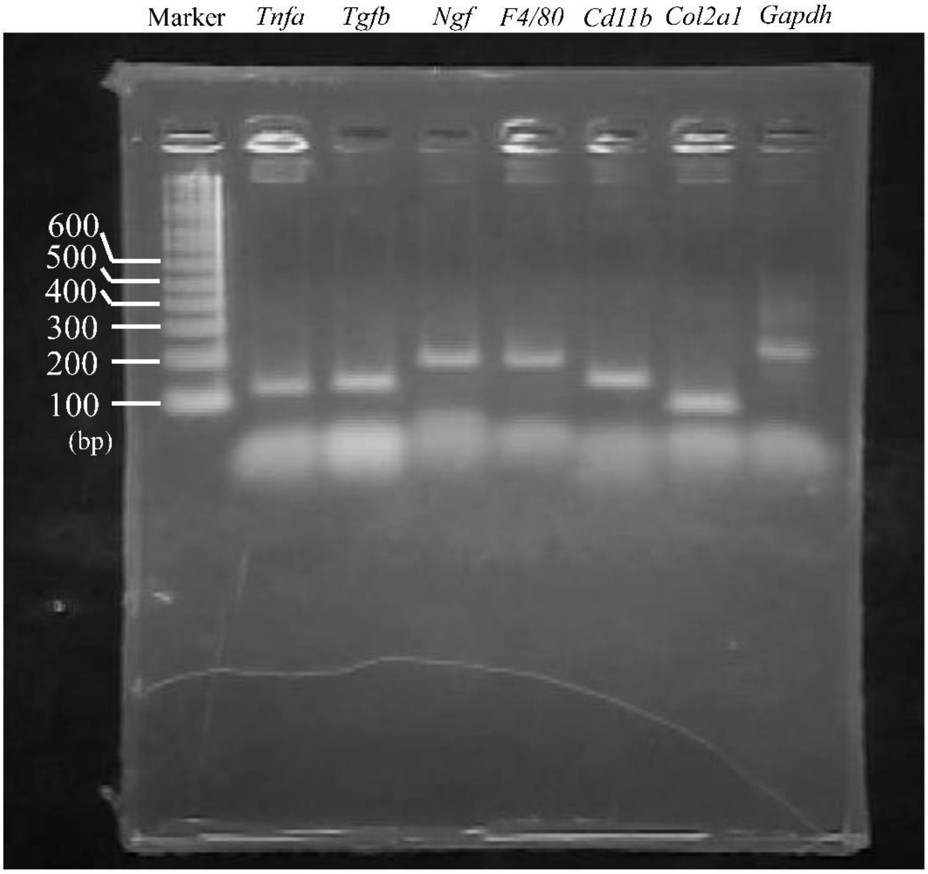

Supplemental Figure 1 Agarose gel electrophoresis of qPCR products
