# Supplementary material for: TGF-β regulates nerve growth factor expression in a mouse intervertebral disc injury model
Source: BMC Musculoskelet Disord. 2021 Jul 23;22:634. doi: 10.1186/s12891-021-04509-w (PMC8299674; doi:10.1186/s12891-021-04509-w)
Supplement: Supplementary file 2 — Additional file 2. [file 12891_2021_4509_MOESM2_ESM.pdf]

Supplementary material 2

To identify a marker of macrophages in injured IVDs, we analyzed IVD cells in injured IVDs at post-injury day 7. IVDs were extracted (n=5), digested with 2 mg/ml type I collagenase for 18 h at 37°C, and passed through a 100-µm cell strained to obtain single-cell suspensions. The disc-derived cells were incubated with brilliant violet 421-conjugated anti-F4/80 (Product no. 123132, Clone BM8, BioLegend, San Diego, CA) and PE/Cy7-conjugated anti-CD11b (Product no. 101216, clone, M1/70, BioLegend) antibodies for 45 min at 4°C. After washing twice in phosphate-buffered saline, 30 000 total events were acquired using FACSVerseTM (BD Biosciences, San Jose, CA), and the data were analyzed using Flow Jo 10.0 (Tree Star, Ashland, OR).

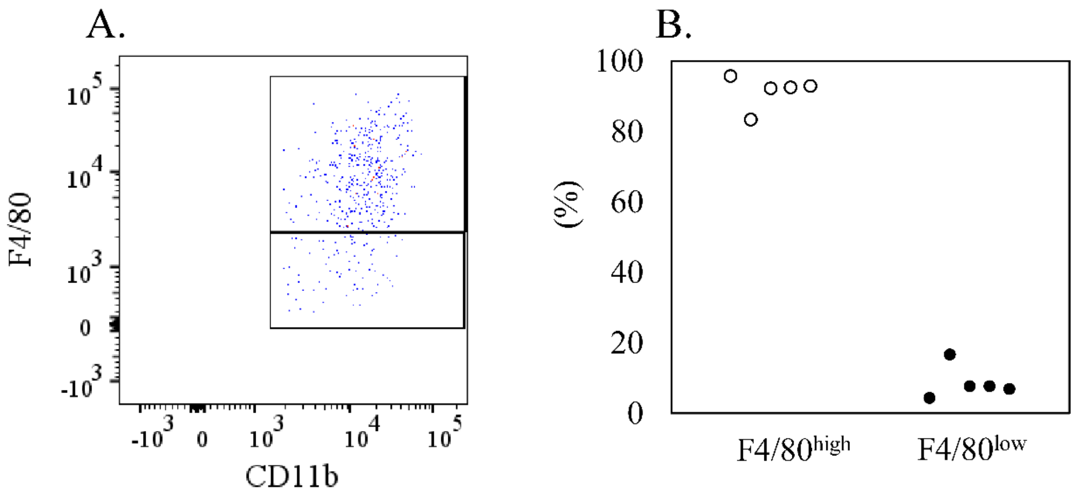

Supplemental Figure 2 Flow cytometry analysis of F4/80high and F4/80low cells among CD11b-positive cells in intervertebral discs at post-injury day 7

(A) The dot plot shows F4/80high and F4/80low cells among CD11b (+) cells in intervertebral discs at post-injury day 7. x-axis: CD11b, y-axis: F4/80. (B) Comparison of the proportion of F4/80high and F4/80low cells among CD11b (+) cells (n=5).
